# Innovative Designs of Multimodal Imaging Based on Radionuclide, Quantum, and Cargo-Loaded Nanoplatform Emitters Towards Enhanced Energy–Matter Interactions for Photonics and Bioassays

**DOI:** 10.3390/ma19163475

**Published:** 2026-08-17

**Authors:** Marcelo R. Romero, Daniela A. Quinteros, A. Guillermo Bracamonte

**Affiliations:** 1 Departamento de Química Orgánica, Facultad de Ciencias Químicas (Universidad Nacional de Córdoba), IPQA–CONICET, Córdoba CP 5000, Argentina; marcelo.ricardo.romero@unc.edu.ar; 2 Unidad de Investigación y Desarrollo en Tecnología Farmacéutica (UNITEFA), CONICET, Departamento de Farmacia, Facultad de Ciencias Químicas, Universidad Nacional de Córdoba, Ciudad Universitaria, Córdoba 5000, Argentina; danielaquinteros@unc.edu.ar; 3 Departamento de Química Orgánica, Facultad de Ciencias Químicas, Universidad Nacional de Córdoba, Córdoba X5000HUA, Argentina; 4 Consejo Nacional de Investigaciones Científicas y Técnicas (CONICET), Instituto de Investigaciones en Fisicoquímica de Córdoba (INFIQC), Córdoba X5000HUA, Argentina

**Keywords:** bio-optics, nano-radio-pharmacy, improved radionuclides, enhanced nanoemitters, multimodal nanoemitters, cargo-loaded nanoparticles, high-intensity electromagnetic fields, nano-optics

## Abstract

**Highlights:**

- Generation of multimodal imaging.
- Application of radionuclides and tracers.
- Cargo-loaded nanoplatforms for multi-functional applications.
- Design of new optical set-ups incorporating enhanced nano-optics.
- Development of nanophotonics towards photonics applications.

**Abstract:**

This mini-review is intended to show how multimodal imaging could be developed from prototypes and proofs of concept by controlling the nanoscale for improved resolution of life science imaging for broad applications such as bioassays, early diagnoses and further applications. It intends to afford the presentation of multimodal approaches for imaging and bioimaging uses with potential applications to biological media. The application of multimodal nanoemitters provides enhanced bioimaging through the generation of various targeted and well-defined signals. Radionuclides and luminescent emitters were considered in the discussion for improved and enhanced signaling. In this manner, we intended to show the increase in the power of information by collecting varied optical signal–matter interactions. This could be important for Positron Emission Tomography and Computed Tomography (PET-CT), Fluorescence Tomography (FT), and other new modes of imaging contemplating the incorporation of nanotechnology. In the context of the design of new multimodal energy modes, key examples were shown from the literature, where the interactions of different energy modes could lead to enhanced and improved signaling. Electromagnetic fields and nanoplasmonics are involved in these different energy modes involving varied quantum particle interactions with modified properties. In this regard, multimodal imaging has experienced developments in nanoemitters and nanobiolabeling to track biomolecular events and targeted cells. A large quantity of research output actually focuses on nano- and quantum emissions. However, there are not as many studies dealing with enhanced emissions or laser emissions coupled with radionuclide emitters. A non-classical form of light emission, considering varied luminescent phenomena as well as further quantum signaling, showed interesting and high-impact perspectives when combined with nuclear emissions. This is the case for current trends focusing on innovative single-cell analysis. For example, the characterization and diagnosis of cells where the targeting of antibody–antigen interactions is required, providing light and energy from different sources to produce different details and imaging resolutions, has been noted. These are potential approaches that could be developed through various strategies targeting life science applications. In this regard, this article puts forward a discussion focused on nanotechnology contemplating radio-pharmacy and enhanced nanoemitters.

## 1. Introduction to New Luminophore and Radionuclide Tracer Approaches

**Figure 1:**
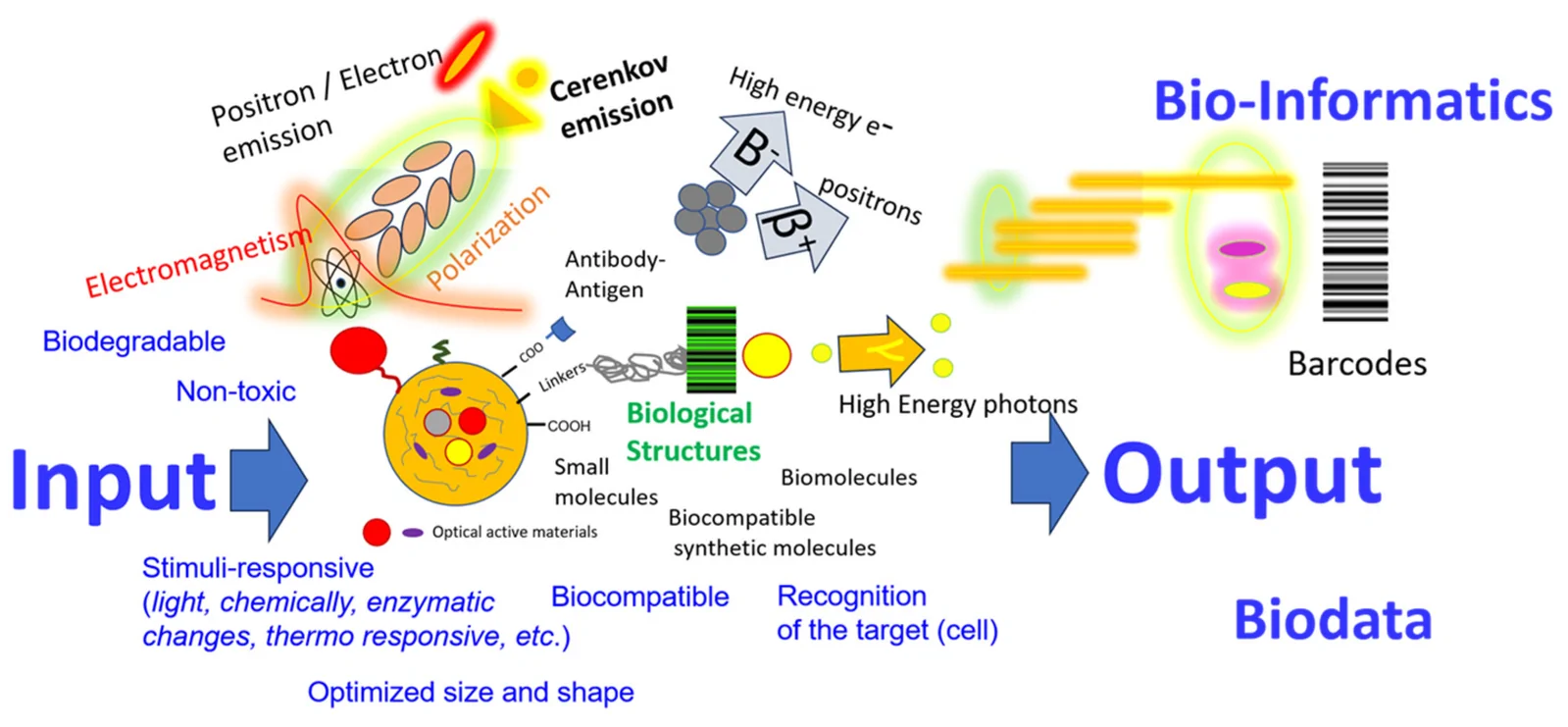
A schema showing enhanced coupling of multimodal radionuclides, luminescence, and further non-classical light emissions. The prototype shows, from left to right, how input data such as a remote laser stimulation could activate optical active nanoplatforms coupled to multimodal energy modes (electrons, positrons, radionuclides, Polaritons, Cerenkov emissions, photons, fluorescence, luminescence, etc.). Thus, electromagnetic fields interacting with different energy levels affecting their emission mechanisms are shown. Red nanoplasmonic spheres show how quantum properties could be tuned and modulated, considering molecules, nanomaterials and larger-sized assemblies as well. The output is associated with multimodal and functional signaling that produces bio-informatics for theranostic perspectives, where detection, diagnosis, precision treatment and tracking of the development of related biological events occur.

This mini-review is intended to show and discuss the basis, insights, and advances of multimodal imaging, including how it could be developed from prototypes to real applications with the potential to transfer to the life science imaging market. It intends to afford the presentation of multimodal approaches for imaging and bioimaging uses with potential applications to biological media. Bioimaging is produced through the generation of various signals from real samples in the form of optical information, or radiation from intrinsic matter. Positron Emission Tomography and Computed Tomography (PET-CT) [1,2], Fluorescence Tomography (FLT) [3], and other new modes of imaging contemplate nanotechnology and quantum technology [4]. In this regard, multimodal imaging has experienced developments in nanoemitters and nanobiolabeling to track biomolecular events and targeted cells and tissues. In this context, currently, the pharmaceutical sector is innovating the development of radio-pharmacy, focusing on targeted tasks involving patient-centered clinical assays. The conception of innovative nanoplatforms incorporating different energy modes to generate imaging could provide multiple resolution levels and develop different optical effects and interferences. The next sections are intended to present many of the mentioned enhanced phenomena. This is just an introduction and invitation to continue forming the bottom-up knowledge required to propose new prototypes (Figure 1). In the design of new confined emitters, the interactions of optical nanomaterial properties such as plasmonics with other sources of energy, such as laser dyes in the form of luminophores and radionuclides, are involved. From these designs, different interactions and effects are shown.

**Figure 2:**
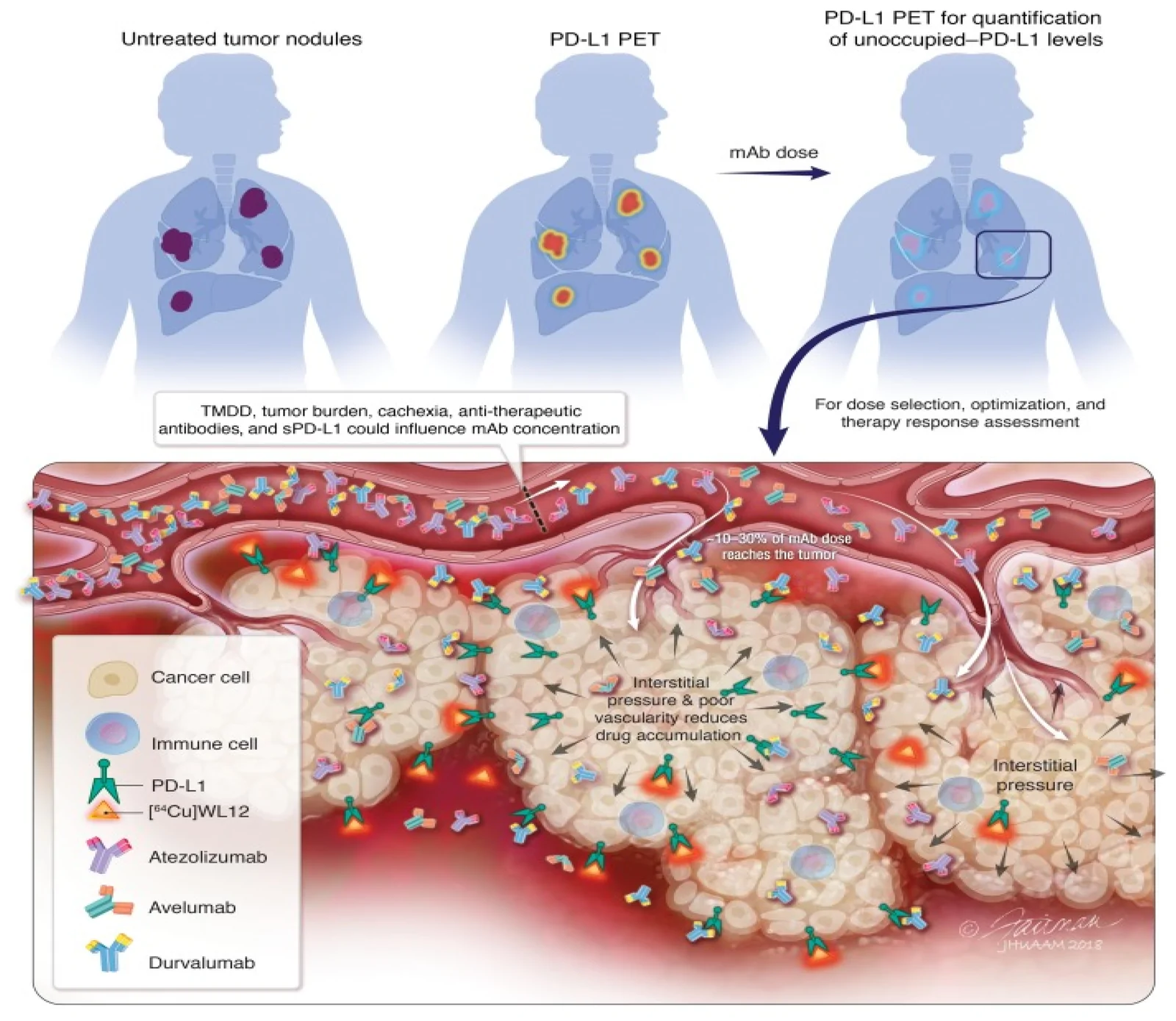
Scheme of doses applied for the diminution of unoccupied PD-L1, in MDAMB231 tumors generated in mice, with increases in the AtzMab dose (mg/kg) incorporated. The AtzMab drug was injected 24 h prior to the radiotracer injection. Thus, PD-L1 PET therapy and evaluation are represented. Notes: sPD-L1, soluble PD-L1; TMDD, target-mediated drug disposition. Reprinted with the permission of the authors: [7] D. Kumar et al. 2019, J Clin Invest., American Society for Clinical Investigation.

Prototypes ideally show enhanced or amplified properties from close light–matter interactions with electromagnetic fields that modify the electronic properties of the quantum particles in focus, expanding images to include everything from the quantum yields of photons to faster ballistic energy paths within complex mechanisms. New enhanced emissions could be produced through the generation of non-classical light involving confined nano-arrays with amplified electromagnetics and with the incorporation of luminescence emitters, semiconductors with photoluminescent emissions, and further emitters beyond the molecular levels towards quantum sizes [5]. Further optical properties could be involved in the mentioned interactions, such as optoelectronics, electromagnetism, varied bosonic particles, and further energy modes such as nuclear energy, radionuclides, etc. In this context, use of the nanoscale within current imaging systems as a strategy to produce electronic modifications within shorter distances is applied to optimize pixel-to-pixel signaling, resolution, and information. However, it is not the only parameter of performance required. Therefore, the following parameters should be contemplated in design to prototype the new associated properties: (i) sensitivity, (ii) spatial resolution, (iii) tissue penetration, (iv) specificity, (v) biocompatibility, (vi) clinical translation, (vii) radiochemical stability, (viii) toxicity, (ix) manufacturing challenges, and (x) regulatory considerations. It is very important to highlight the factor in common and the need to study and develop new multimodal light–matter interactions. In this regard, and in accordance with biosafety measures, multimodal imaging is combined with varied materials and bioassays daily in fundamental studies, driving breakthroughs in theranostics. However, not all of these could produce acceptable results on this important issue that could transfer to a real application. Thus, by using the same molecule for both diagnosis and targeted therapy, high-precision measurements such as transitions from beta-emitters were recorded. In this regard, as an example, Lutetium-177-targeting alpha-emitters as well as Actinium-225 and Lead-212 were used [6]. Incorporating these perspectives, applied studies involving radiation–matter interactions generating targeted resolutions and bio-interactions are in progress. In the literature, there are many innovative applications highlighting immune profiling as a biodetection strategy. Another example that could be mentioned is a class of cancer treatment that blocks checkpoint proteins acting as “off switches” for immune T cells; however, the related techniques are still focused on improvements and early diagnoses accompanying the tracking of treatments. In order to achieve this level of design, the multi-functional and multi-modal energy approaches are considered. Therefore, based on PET imaging, the application of peptide-based radiotracers of [64Cu]WL12 in vivo was reported to study trafficking and distributions using multiple xenograft models. In this manner, the capability of tracking improved pharmacokinetics of antibodies from variable PD-L1 expressions was shown (Figure 2) [7]. Therefore, an inhibitory sigmoidal Emaximal model was produced that appropriately fit with the experimental data recorded. The application of AtzMab correlated accurately with the decrease in unoccupied PD-L1 in tumor sites, where AtzMab is the Atezolizumab Monoclonal Antibody used for immunotherapy drugs that are commercially available to treat metastatic cancers. So, it is highlighted as an innovation applied to commercial drugs within multimodal and functional approaches to target multiple tasks and record different signals.

Improvements in enhanced radionuclide therapy via the conjugation of varied biomolecules with complex formulations are in progress. In this manner, improved peptide pharmacokinetics showed enhanced therapeutic effects based on peptide–radionuclide conjugates. The covalent conjugation of fatty acids as albumin binders slows blood clearance and produces higher tumor uptake. The incorporation of additional biomolecules in carriers modified the dynamics and interactions to pass through cell membranes. Using this strategy, drug bioconjugates of Pentadecanoic Acids (PD) with 225Ac- and 177Lu-labeled RGD peptides were designed in order to evaluate albumin binding [8]. In this manner, the afforded lipid conjugation increased tumor uptake. As mentioned previously, the rate at which the body removes a substance from the blood, measured as volume per unit of time, was longer, permitting specificity and preferential therapeutic efficacy. So, it was shown that PD-K(225Ac- DOTA)-c(RGDfK) enhanced the targeted radionuclide-based therapy results, improving the tumor uptake. And logically, drastic tumor growth inhibitions were generated. This alone lead to a longer median survival time. So, small biomolecular structures had an important impact at the micro and macro scales within life sciences involving the development of enhanced phenomena related to drug, biomolecular, and nanoparticle trafficking with biocompatible and attractive directional, targeted interactions. However, even within the smaller scales, current challenges related to opto-electronics interactions involve enhanced and further coupling phenomena. In this regard, emissions with different functions could be involved.

Considering enhanced performance and improved quantum yields, challenging themes and related topics appear regarding new topological states of light, where confined electronics at the quantum scale produce stable quantum dots. In addition, nano-physics at the quantum scale coupled with nanoplasmonic phenomena could produce greater enhancements. By leveraging the Purcell effect—where metallic nanostructures alter the spontaneous emission rates of quantum emitters via confined electromagnetic fields—researchers are drastically improving both the sensitivity of radiation detection and the efficiency of targeted radiotherapy [9].

Moreover, generating non-classical light at the nanoscale relies on confining light–matter interactions to sub-wavelength dimensions. For example, by using quantum dots as solid-state quantum emitters with tunable colors based on diamond/SiC materials, quantum structures within 2D and 3D images were developed further. Therefore, from films to nanophotonic cavities, it is possible to tune triggered single photons and entangled photonic pairs. This fine and high-sensitivity photonic resolution and control was, over time, produced by controlling quantum photonic purity, which involved the absence of interferents, energy losses and even coherence [10,11]. So, this was afforded to new light–matter interactions and properties in synthetic solid matter and labeled tissues from real samples. Otherwise, it is also possible to couple optical gain media for the transfer and flow of non-classical light. In this context, and to show the complexity and capability of transferring quantum energy, the development of waveguides for the flow of non-classical light produced by Herald electrons is noted [12]. From this, it is possible to understand the broad implications and potential development for advanced imaging. So, the generation of new quantum approaches, e.g., those based on film depositions on tissues or special colloidal formulations, could represent the next generation of light-based imaging technologies. Therefore, the intended multimodal approach in design and synthesis should not be forgotten. In fact, a large quantity of output is focused on the tuning of nanoscale and quantum light in order to incorporate alternative and new energy modes depending on the needs of the real samples in focus. However, there are not many studies dealing with enhanced emissions or laser emissions coupled with radionuclide emissions.

Non-classical light emission coupled with nuclear emissions could lead to early diagnoses and analyses from different perspectives on the same problem, providing multimodal energy approaches. For example, this is the case in the characterization and diagnosis of cells where the targeting of antibody–antigen interactions is required, providing light and energy from different sources to produce different details and imaging resolutions. These are potential approaches that could be developed for various strategies targeting life science applications. Thus, a discussion was developed focusing on nanotechnology, contemplating radio-pharmacy and enhanced nanoemitters for nanophotonics, biophotonics, bioimaging and nanomedicine applications involving new enhanced phenomena as strategies to augment the power of information and capabilities in the analysis, diagnoses, and further decisions applied. In this regard, the growth of brain tumor cells was studied in vivo via bioluminescence and Fluorescence Tomography imaging, including follow-ups (Figure 2) [13].

**Figure 3:**
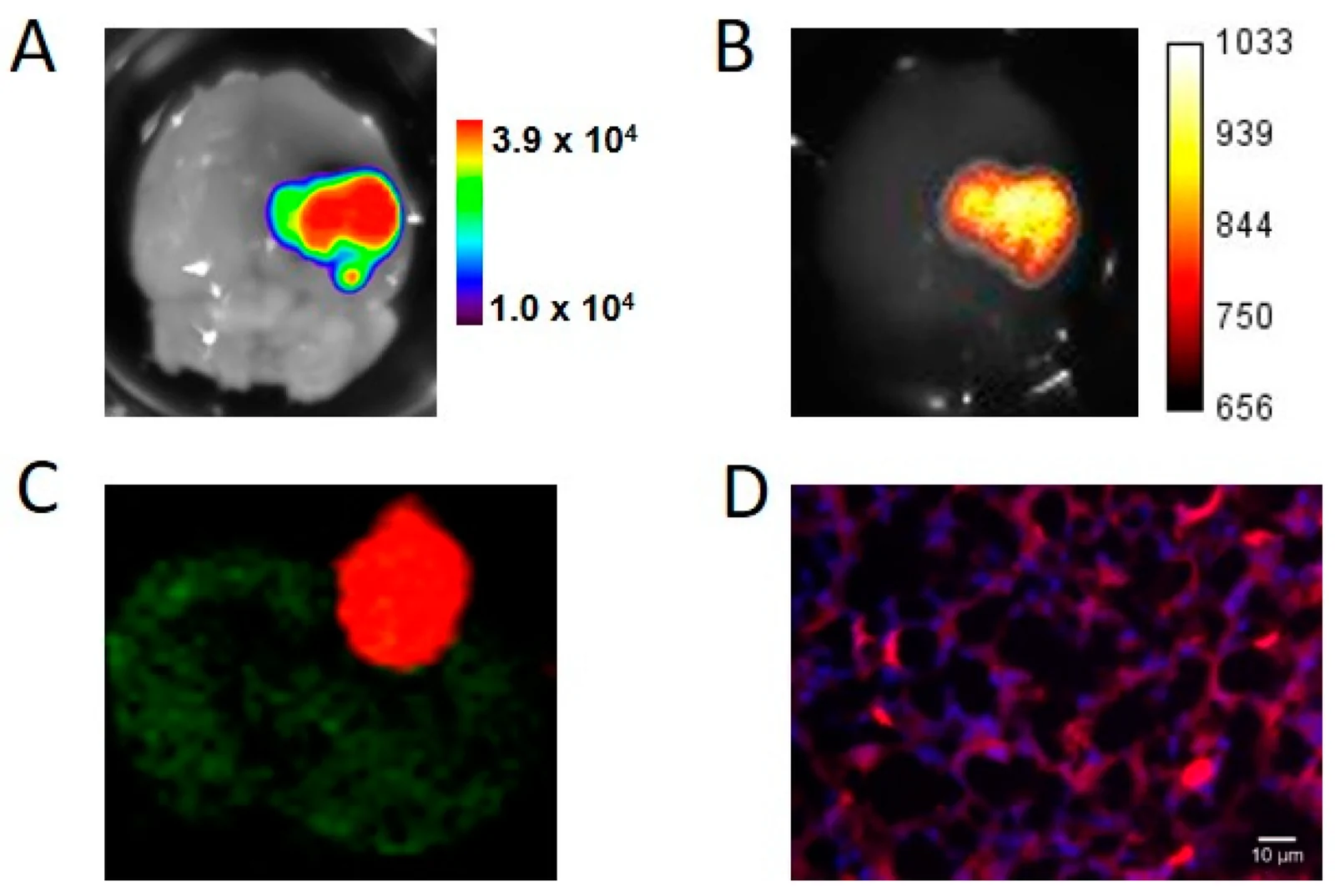
Cell imaging of postmortem rodents expressing U87-iRFP+-Fluc+ in the brain. An excised brain was sequentially imaged by BLI (**A**) and FRI (**B**) to reveal tumors. (**C**) Cryosection of the brain tumor was scanned for fluorescence using the Odyssey scanner showing iRFP fluorescence at 700 nm (red) and brain autofluorescence at 800 nm (green). (**D**) Fluorescence generated by iRFP (red) and the nucleus (DAPI, blue) was detected by Fluorescence Microcopy with standard xenon lamp excitation. Reprinted with the permission of the authors; citation: [13] Coralie Genevoise, Huges Loiseau, and Franck Couillaud et al. 2016, Int. J. Mol. Sci., MDPI.

The researchers were able to track cancer cell growth from brain imaging, highlighting the use of images based on the generation of the bioluminescence of firefly luciferase (Fluc), which was used as a gene reporter, while d-luciferin was used as a substrate (Figure 3A,B). After that, luciferin was injected into postmortem rodents to track brain bioimaging. Thus, they were able to detect different brain states during the development of cancer cell growth. The capability to generate phenomena associated with different functions in the context of diagnosis and therapy for important health problems was highlighted. In this manner, the researchers were able to remove unhealthy cells sequentially using BLI for enzymatic activity and FRI for near-infrared fluorescent protein (iRFP) fluorescence from imaging analysis. The images showed very good correlations between the U87-iRFP+-Fluc cells and tumors. These correlations and the prototyped cell treatments advanced future high-precision medicine. It is mentioned that the experiments were well organized and pre-designed to control for different expressions. Thus, U87 cell lines were genetically engineered to express encoded genes based on Fluc and iRFP reporters in cells, subcutaneous tumors, and the brain. In this manner, the researchers were able to detect targeted modifications by fluorescence imaging.

Moreover, the tumors were fixed and frozen to afford fluorescence from iRPP in U87-iRFP+-Fluc+ tumor cells. For these studies, the Odyssey scanner and epifluorescence microscopy were used (Figure 3C,D). This approach was chosen to record various signals in looking for different specific targeted objectives.

In this manner, as was discussed, the incorporation of varied sources of energy, including radionuclides, quantum emitters, fluorophores, and even new non-classical light sources from radiation–matter interactions, provided interesting insights and innovation in the biodetection of targeted biostructures such as cancerous cells. In a similar manner, bioconjugation showed other types of enhanced perspectives related to improved and higher incorporation within cells; therefore, detection and treatments are enhanced as well. From these perspectives, the next sections are focused on the different aspects involved, highlighting their uses. The enhanced perspective from light–matter and electronic interactions could provide improved signaling, including switching between different resolutions, and imaging information. Moreover, the enhanced concept could also be applied to the delivery of radionuclides for faster and highly accurate targeted treatments. This involves different challenges related to multiple factors, functions and media. Thus, the enhanced delivery of radionuclides at the nanoscale leverages sophisticated material engineering to improve cellular uptake, maximize radiation retention in tumor tissue, and minimize off-target toxicity [14]. Both effects could be present in the same approach; however, they occur through different mechanisms.

## 2. Enhanced Nanoemitters for Biophotonics

In this section, the development of optics typically refers to optical systems, materials, or components that have been upgraded to break classical physical barriers and deliver and focus with higher energies whilst involving reduced sizes, such as those from the quantum and nanophotonic scales.

The innovation of small and ultrasmall tunable nanoemitters revealed an unsolved paradigm in optics and plasmonics phenomena below and beyond the nanoscale. Quantum dots with sizes below 10 nm showed strong emissions at variable wavelengths [15,16]. But modified plasmonic surfaces enhanced their emission properties based on Metal-Enhanced Fluorescence (MEF) [17]. This phenomenon was explained by enhanced excitation by the emitters within the near, highly electromagnetic field of metallic nanoparticles [18,19,20]. In this manner, metallic core–shell nanoparticles showed high emission enhancements accompanied with diminished photobleaching properties due to accurate tunable 50.0 nm silver nanoparticles and silica spacers [21]. In addition, multilayered fluorescent silver core–shell nanoparticles showed improved enhancements, developing Fluorescence Resonance Energy Transfer (FRET) [22] coupled to MEF [23,24,25] that permitted the ultra-low DNA detection of different human genes [26]. Moreover, 40.0 nm gold core–shell nanoparticles showed ultraluminescent properties [27], even if their plasmonic intensities were lower than silver nanoparticles due to their intrinsic electronic properties. Then, these ultraluminescent properties were applied for bacteria labeling and individual biostructure detection [28].

The generation of controlled emissions associated with specific frequencies, photon and lifetime decays, wavelengths, energies, and various colors could be delivered through space and time via various techniques. However, it should be highlighted that these emissions are from controlled tiny particles within the nanoscale, which in many cases are closer in size to those at the quantum scale. This means that particles sized 1 nm and beyond could be high-intensity emitters that deliver illumination, producing different images and resolutions depending on the real sample tissues and optical active components that are intrinsically and naturally incorporated. And, in this context, by highlighting the resolution and applied effect on the tissues and cells, it possible to tune the delivery of energy modes depending upon the needs and intensities. In this manner, the delivery of photons, for example, could be controlled, providing time delays between different biological phenomena and the coupling of interests. In a similar manner, the energy could produce further targeted effects such as heat or biomatter degradation. So, it is noted that these other effects in signaling are applied for imaging generation or targeting biomolecules.

Thus, enhanced techniques, including methods such as MEF, are under research and investigation, with an eye towards technological transference. Regarding neophytes, LED lights and mobile phones already incorporate metallic and electromagnetic field generations within specific contacts for enhanced and brighter light production. In this regard, within the nanoscale and life science applications, silica-based nanophotonics could be highlighted for various enhanced strategies, such as tuning non-classical light FRET delivery [29]. In this manner, for cyanobacteria, enhanced fluorescent silica nanoparticles were deposed on biostructures. Therefore, bioimaging was generated from the synthesized nano-biostructures through fluorescence microscopy using laser excitations. Cyanobacteria labeled with SiO_2_-RhB (Rhodamine B: RhB) nanoparticles did not produce well-defined bioimages of small nano-biostructures. This effect was produced only when SiO_2_-RhB nanoparticles were deposed on cyanobacteria, since free nanoemitters showed their non-quenched emissions. Additionally, with fluorescent mono-colored SiO_2_-Fl nanoparticles, bright, clear and enhanced labeled cyanobacteria emissions were obtained. Yet free nanoemitters such as SiO_2_-Fl nanoparticles showed lower emissions than SiO_2_-Fl-labeled cyanobacteria.

However, cyanobacteria labeled with higher intensity SiO_2_-RhB-Fl emitters generated low-emission nano-biostructures, whereas non-labeled biostructures, as already reported [30], showed weaker emissions, producing bioimaging with lower resolution. This observation could be tuned by varying the relations of dyes and the concentrations incorporated; however, in comparison to free nanoemitters, the emission intensities of biostructure emitters were always lower. This fact was explained in part by the lower quantum yields and quenching electron transfer occurring in PSII [31]. Retro electron transfer (RET) in cyanobacterial Photosystem II (PSII) involves the backward flow of electrons, often from the quinone acceptor site back towards the reaction center, especially under stress (like high light or salt) or to balance energy, preventing damage by reducing over-excitation or facilitating energy dissipation as heat (non-photochemical quenching) rather than full charge separation.

Therefore, light delivery was afforded by tuning Fl dye concentrations and coupling PSI to optimize the results in these conditions. The depositions of the different nanoparticles on cyanobacteria were corroborated by TEM images.

Later, it was corroborated that these mechanisms became involved by varying the cyanobacteria species and photosystems incorporated. In the next section, the different targeted natural chromophores within the biostructure and resolutions of synthetic non-classical light are shown.

The mentioned enhanced luminescent properties showed a high impact in biophotonics applications, such as for fluorescent imaging in vivo [32]; however, for applications within cells, the resolution should be lower and below the micro-scale for organelle labeling and biosensing within the biostructure. These types of applications were reported in immunoassays and fluorescent labeling [33]. At this point, it is important to highlight the effects of materials, sizes, geometries, biosurface interactions and the biocompatibility of nanomaterials for their incorporation [34,35,36]. Moreover, the application of FRET for biological event detection and tracking permitted, for example, molecular detection within cells and cellular cancer tracking [37], which was shown to be largely applied by Fluorescence Lifetime Imaging Microscopy (FLIM) [38]. So, the design of nanoplatforms of a reduced size for enhanced FRET in imaging at quantum and nanoscales is of high impact for a huge number of biophotonics applications in Advanced Precision Nanomedicine Diagnostics. In particular, the 3D resolution and spatial sensitivity of FRET to the positions of donors and acceptors involving antibody–antigen interactions and immunology reactions are highlighted.

In this regard, it should also be highlighted that nanoplasmonics could produce improved effects on radionuclide emissions. As an example, radionuclide–Au nanoplatforms were synthesized and conjugated with DNA for biocompatible radiolabeling chemistry. This multilayered Au shell combination enabled higher radiosensitivity and excellent in vivo stability for enhanced dendritic cell-based cancer immunotherapy. In this case as well, the nanoplasmonic phenomenon shows spatial sensitivity that could produce variations within the known near fields involving shorter distances at the nanoscale and is closely related to the immunological phenomena and dynamics afforded in biological systems of interest. In this case, it was highlighted that the Cerenkov luminescence was accompanied by PET to afford quantitative tracking of dendritic cells [39]. Moreover, it is important to highlight that the mentioned enhanced Cerenkov luminescence emission is generated at a different excited level of energy, with a pale blue glowing emission produced by beta-emitting radionuclides, like those used in PET scans [40]. The emitted radionuclides move through a liquid gain medium faster than the speed of light. This fact allowed for a wide spectrum of applications to be proposed within the life sciences. Thus, enhanced radionuclides and luminescence represent the synergistic use of radioactive emissions, such as beta or alpha particles, to stimulate a glowing optical signal. Regarding advantages, the capability to apply controlled dosimetry and remote detection is highlighted. Thus, applications such as Radionuclide-Activated Luminescence for Cancer Theranostics could be found [41]. It is very important to control these high energy levels with targeted biodetection and therapeutics. In recent years, the convergence of enhanced luminescence, nanoplasmonics, and radionuclide interactions has driven significant advancements in radiotherapy and radiodosimetry. This combination provides a new direction towards specific solutions in precision oncology for destroying tumor cells. However, the need to track doses of radiation produces interest in a new design on a tiny scale, combining synergy-specific and enhanced energy mode productions and controlled delivery even more. Thus, by combining the physics of light, metallic nanostructures, and nuclear decay, this could overcome long-standing limits in both targeted radiotherapy and localized radiation dosimetry, accompanied with more controlled delivery.

The challenge under study is nanoengineering architectures and novel nanocomposites by tuning optical gain media depending on the targeted amplified signal. Therefore, it is possible to amplify optical signals, improve radiation detection, and accelerate bioimaging breakthroughs from enhanced resolutions with higher intensities and sensitivities based on switching between varying excited state levels, such as those stated for radionuclides and laser dyes [42].

In a similar manner, nanoplasmonics and lanthanide complexes near metallic nanostructures, like silver or gold, could strongly couple their emission with surface plasmon resonances, generating faster radiative decay rates and higher emission intensities. Enhanced up-conversion photoluminescence (UCPL) occurs due to electronic density modifications promoted by electromagnetic fields, improving radiative emission paths. In this manner, the sizes, shapes and materials could be tuned to couple ultrafast mechanisms involving different variables depending on the intrinsic matter constitution of emitters. However, it is known that, in general, the emitters could improve their quantum yields by augmenting their absorbances and consequently their electronic excited state occupancy. However, there is not a well-defined rule for the known phenomenon because there are multiple variables affecting the enhanced phenomena. Thus, the variable shapes and materials involved could produce different optical gain media due to the targeted enhancements. For example, the precise tuning of surface plasmon resonances within complex epitaxial and asymmetrical 3D structures, controlling relations between silicon-based NaGdF_4_:Yb^3+^, Er^3+^ nanoparticles and gold dendrites, produced effective plasmonic enhancement of up-conversion photoluminescence [43].

From these perspectives, and considering the incorporation of further nanophysics and nanochemistry with varying impacts on imaging, in the next section, we present the current methods using radio-labelers and tracers with potential interactions with further nanomaterials and their associated phenomena in order to develop multimodal imaging approaches.

## 3. Current Imaging Methods Highlighting Molecular Labelers and Tracers for Early Diagnosis and Medicine

In this section, we show the current imaging systems with advanced optics available in clinics, hospitals, and research centers that apply various prototypes of nanotechnology in the pursuit of enhanced optical properties and information. This was intended to show the enhanced properties developed through the incorporation of the new prototypes and formulations applied. The enhanced delivery of optical signaling and drugs depending upon need identifies potential applications and further developments. Enhanced properties are used to discuss, display and propose new strategies. In this context, enhanced radionuclide delivery leveraging electronic complementarity is an advanced engineering strategy in radio-pharmacy that utilizes matching surface charges, dipole moments, and electrostatic potentials between a delivery vehicle and its biological target to maximize diagnostic and therapeutic outcomes. As an example, and to highlight the importance of the biocompatibility and improved delivery of radionuclides, the higher pharmacokinetics recorded due to the application of peptide cyclization is noted. In brief, cyclization increases the pharmacokinetics, optimizing the levels of the energy barriers involved to fit peptides in the membrane media and develop transport of proteins. Thus, passive diffusion or an active spatial distribution change across cellular membranes is facilitated. In this manner, enhanced absorption and membrane permeability were produced [44]. This capability, combined with radiative materials, permits the improved delivery of highly sensitive optical response materials such as radiotracers. In addition to controlled delivery after overcoming biological constraints, the potential is provided to activate delivery at the right place, and ideally facilitating the respective enhanced phenomena as well. These bases and the related challenges, afforded from years of study, show that the delivery of radiotracers in their free forms was not possible for various reasons, from solubility to toxicity issues. So, the design and synthesis of new multimodal systems was shown to be of high interest. In this regard, many other examples from fundamental sciences incorporating enhancements are under discussion as well, involving electronic densities and electromagnetic fields [45]. Thus, meticulously matching the local electron density of the targeting vectors, such as small molecules, cyclic peptides, or nanocarriers and even nanoparticles, with the complementary electronic environment of the targeted receptor could enhance more than light pathways. In addition, ballistic-improved complementary paths could be generated. These are challenges and strategies that are intended to showcase current research and further developments. 

There are many imaging techniques and methods under development; however, not many on the market can be completely transferred to life science uses. It should be noted that many well-known imaging methods exist for clinical diagnosis where non-ionizing radiation is applied. Most of them are non-invasive and provide good resolution and details of the whole human body. But they could resolve the varying image dimensions depending on the technique and energy mode applied. Thus, multimodal approaches are needed from a practical point of view, with different perspectives from different optical information recorded. In this context, magnetic resonance imaging (MRI) is mentioned as a very well-known technique; it uses images for medical use [46] without involving ionizing radiation and provides very good resolution of living tissues and organs. These characteristics provided by MRI have widespread uses and real applications in clinical imaging. Moreover, Positron Emission Tomography (PET) is used in oncology, neurology, and cardiology to measure vital cellular functions, metabolic activity, and blood flow using safe radioactive tracers [47]. In particular, for this technique a specific reagent should be ingested to generate signaling. Thus, it requires the ingestion of Fludeoxyglucose F18 (FDG), which acts as a radiotracer agent that emits positrons detected by the PET instrumentation. This technique provides high resolution and detailed imaging for early diagnoses and the monitoring of variations in health status, such as malformations, new tissue production, brain state, etc. [48]. However, it is known that it is not very useful for staging in gastric cancer, largely due to the fact that most gastric adenocarcinomas have low fluoro-deoxy-glucose uptake. This technique could be coupled with Computed Tomography (CT), another imaging technique. It consists of larger-sized cylindrical instrumentation, allowing the human body to enter and be scanned in order to record signals to be processed with appropriate mathematical models.

PET imaging with multimodality configurations has emerged in combination with Computed Tomography (CT) and, more recently, with MRI. In this manner, it is possible to achieve targeted imaging with clear organ resolutions. Tracers are used to show chemical changes that occur in unhealthy cell states, while structural scan maps of body parts are paired with computing methods.

**Figure 4:**
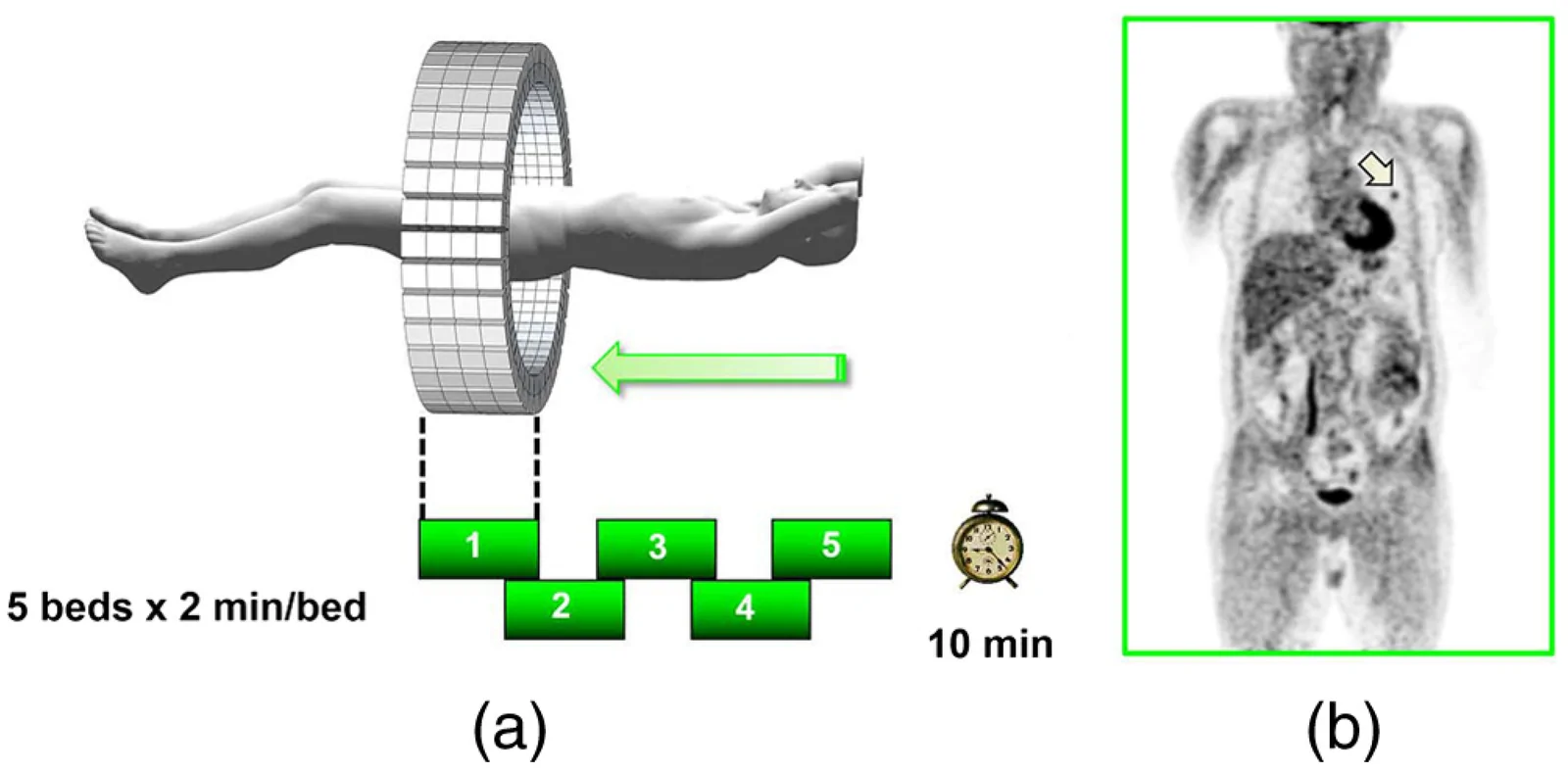
Scheme of PET imaging system with a patient to record the targeted FDG deposited in particular regions related to excess sugar production. The system incorporated a multi-position bed to scan and overlap different segments of the body: (**a**) the bed covers 5 to 7 positions, depending on the height of the patient, to acquire the image; (**b**) the labeled glucose in all organs throughout the body. In this manner, a tumor is shown in the region of abnormal uptake, at the apex of the lung, indicated with a white arrow. Reprinted with the permission of the authors; citation: [49] Terry Jones, David W. Townsend 2017, Journal of Medical Imaging, SPIE.

It is possible to scan the whole body using the cutting-edge technology of PET/CT (Figure 4) [49], occurring in under 10 min for clinical applications. Thus, the imaging produced by the injected FDG affords stable recording while the bed moves in discrete overlapping steps. In this context, variables beyond the focus of radiation are involved, considering different positions from the base of the brain to the thighs in five to seven bed positions, depending on the height of the patient. And focusing on the molecular level, the detection of glucose in all organs throughout the body is possible by using the appropriate bioconjugated tracer. In this context, the biodetection of tumors and abnormal uptake of tracers are highlighted within images from the apex of the lung of a patient.

It should be noted that PET imaging resolution is improved by combining it with CT scanning at the same time [50]. This enhanced resolution is based on the molecular tracer interacting within the biological media within confined nano- and microscales, such as those involved at the biomolecular level of modified proteins and organelles. Thus, PET scanning is currently applied to the early diagnosis of cancer, blood flow analysis and evaluations of organ functions. However, there is still a lot of ongoing research focusing on enhanced signaling and specificity within a broad overview of malign cells as well as the biomarkers related to different health states [51]. In this manner, it is possible to sense different dimensions through biological media. So, in brief, by applying PET imaging measures, cellular biosensing targeting metabolism and biochemical activities at a macroscopic scale across the entire body is possible, while OCT captures ultra-high-resolution images within tissues, including cross-sectional structural details, with a layer-by-layer analysis using light waves focusing on specific hot spots of interest.

Moreover, other energy modes are noted, such as the light scattering produced by optical coherence tomography (OCT) [52], which is a technique based on images generated through the use of interferometry with short-coherence-length light. These signals are able to interact deeply at the micrometer level. Moreover, OCT uses transverse scanning of a light beam to form 2D and 3D imaging based on the light reflected and scattered within biological tissues.

Even though all of these techniques are currently in use for real diagnoses, there is still interest in applying varying optical approaches in order to record different levels of information. Multimodal approaches could address this problem and find an accurate and higher-precision methodology. Thus, the development of new imaging agents could overcome certain constraints and exploit the incorporation of more than one energy mode.

In this regard, there is huge interest in new developments at the nanoscale molecular level in order to optimize the delivery of varied energy modes, including highly sensitive and specific nuclear energy for various targeted treatments. In a similar manner, there is a well-defined trend of applied technologies based on non-classical luminescence phenomena. Near-infrared fluorescence imaging (NIRF) [53] is applied in real cases, such as surgical interventions requiring a higher resolution of tissues. Therefore, the most well-known NIR fluorophores available commercially could be introduced, and based on these examples, they focus on information and improvements related to imaging-based surgery. The optical tissues’ characteristics are very important variables that could affect the results. In this context, other aspects to consider include quantum yields, stability in the media, photobleaching, and the targeting of properties to the applied tissue. Thus, sources of NIR energy modes are highlighted, such as those from fluorophores and meta-fluorophores, as well as bioconjugated quantum dots with potential uses in labeling and multimodal approaches, seeking to resolve different challenges within the same image when assisting a surgical event. Therefore, NIRF imaging requires the incorporation of contrast agents with fluorescent characteristics in the near-infrared interval of 700–900 nm. Given that this technique is considered minimally invasive, it possesses great potential for clinical practice [54]. In addition, it should be noted that Fluorescence Molecular Tomography [55] makes possible the addition or targeting of natural and synthetic emitters within tissues, as well as the generation of well-resolved imaging below the interval of lengths achieved by previous techniques. However, the existence of all available techniques does not mean they are applied in all possible cases; only one technique is used. This fact supports the development of multimodal points of view as well, which, in real cases, are used when asking for different imaging techniques. This technique is opening the door to more bioassays, such as in vivo and in vitro tests, as well as applications showing high sensitivity. However, challenges related to resolutions, limits of detection and quantification, and photobleaching and interference with tissues are still under discussion.

In this way, the bottom-up approach of enhanced multimodal nanoemitters began to be presented within multifunctional nanoplatforms that integrate multiple diagnostic signals into a single optical system able to track and combine multiple modes of imaging depending upon need. Therefore, by combining emitters like radioisotopes, fluorophores, or magnetic cores, the limitations of single-modality imaging were overcome. In this manner, high-resolution anatomical and molecular mapping for tomography has been reported [56]. In order to interact with varying optically active and non-active biomaterials, it is possible to study various types of nanomaterials and quantum materials as well. Even carbon-based materials have been reported with different and interesting performances related to contemplating control of the quantum scale, as was previously shown. However, at this stage of development, further functions activated at the same time are proposed, such as those developed by graphene oxide-based systems facilitating subcellular targeting and synergistic and innovative therapies for targeted cancer treatments [57].

**Figure 5:**
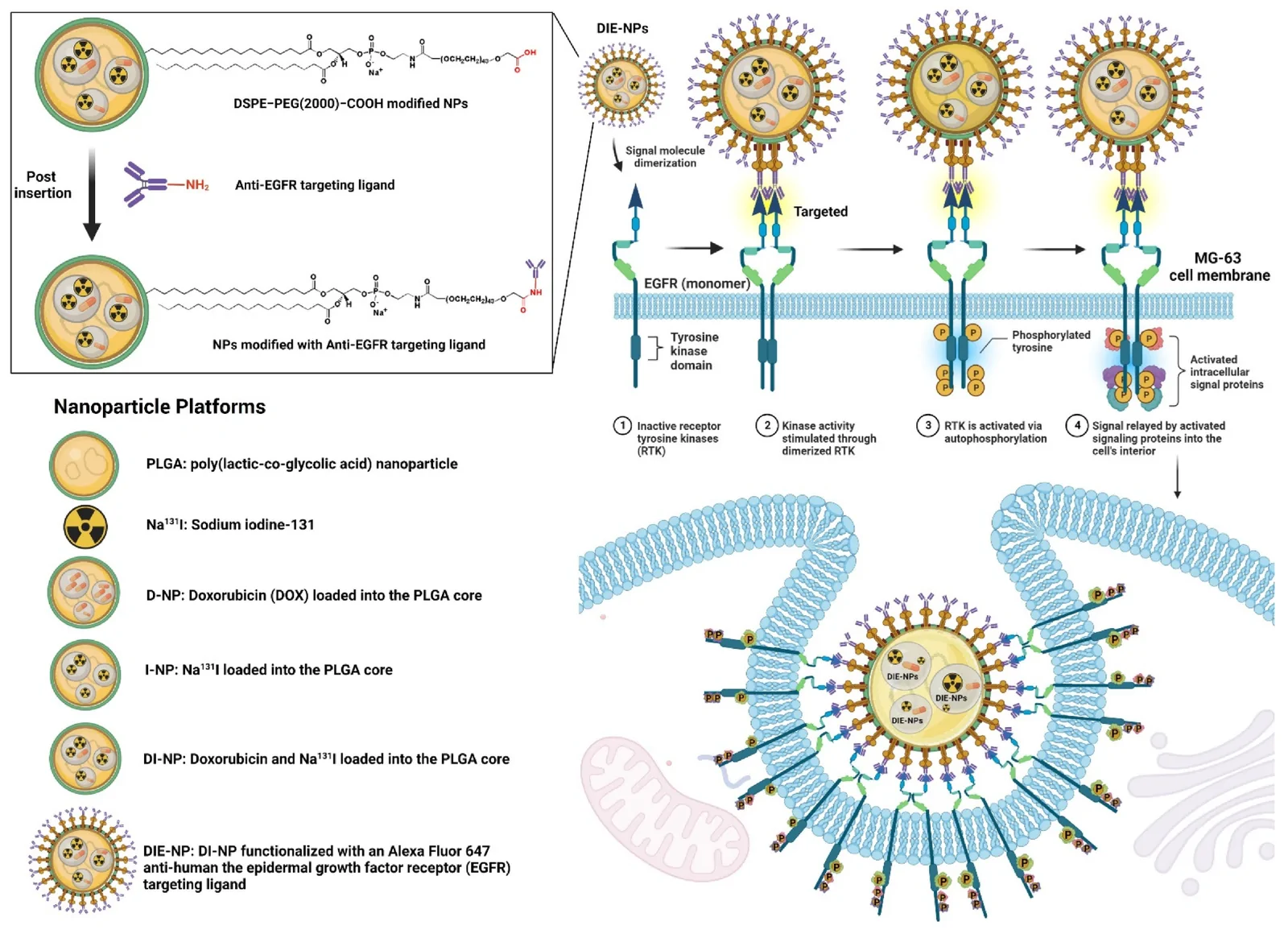
Scheme of DIE-NP nanotherapeutic system interacting with membranes. The fabrication of DSPE–PEG(2000)–COOH-modified DIE-NP, which was modified with an Alexa Fluor 647 anti-human epidermal growth factor receptor (EGFR)-targeting ligand. Published with the permission of the authors; citation: [60] Khamruang Marshall et al. 2022, Nanomaterials.

In this manner, the development of new imaging techniques with improved characteristics, higher versatility, and resolution of new targeted structures is in focus. For example, enhanced optics for imaging molecules, tissues, and living organisms is in progress, from fundamental research stages to technology transfer [58]. In this context, all new optical platforms from quantum, nano-, and micro-scales are welcome when they can add insights and the required improvements. Therefore, from the literature, rapid isotopic exchange in nanoparticles could be mentioned as an example [59]. This study showcased controlled 107Ag/109Ag clusters in a similar manner to H_2_O/D_2_O pairs, which could rapidly exchange within the nanoscale. This spontaneous process was governed by the entropy produced during mixing that involved events within different, simultaneous time scales. Experimentally, concentrations varied around 1.5 × 10^−3^ mM, with varying molar ratios, and were monitored by ESI MS. In this context, the methodology was easily applied with proper instrumentation with high-precision resolution of isotopic exchanges in particular conditions and atoms. This showcased how confined spaces with different energy mode interactions produced signal modifications with potential clinical translations. In a similar manner, biomolecule incorporation in close contact with nano- and quantum platforms could be proposed to modify signaling depending on their structures and electronic densities, along with different health states. This is the concept of advanced studies in many cases. In this regard, another example of controlling conditions within the nanoscale could show similar trends and high-impact insights within bioimaging and clinics. New therapeutics should be noted, such as targeting anti-EGFR using a multifunctional I-131 radio-nanotherapeutic prototype in a new approach to osteosarcoma treatment (Figure 5) [60]. In this study, an important anti-cancer drug, DOX, was encapsulated and joined to Na131I in the inner core, while on nanosurfaces it was conjugated with an epidermal growth factor receptor (EGFR) antibody. Thus, in brief, a bottom-up approach was designed where different properties and applications were combined, with interesting multimodal insights. In this case, it was noted that the co-administration of DOX and Na131I (DIE-NPs) disrupted DNA repair and generated free radicals, resulting in cell apoptosis activation. Furthermore, the PEGylated anti-EGFR functionalized nanoparticles were biocompatible with red blood cells. In this manner, adverse effects were not recorded.

Moreover, additional properties of interest, such as magnetism, could be added with direct impacts on well-known techniques such as MRI. The use of magnetic nanoparticles has been reported for both MRI and PET [61]. In this regard, applications were reported showing improved resolution from nano-contrast agent bioconjugation [62]. This approach improved the high PET sensitivity and high spatial resolution of MRI too. Moreover, perfect spatial registrations of molecular/functional PET and anatomic/functional MR were achieved from these types of magnetic nano-approaches. Both are so important that highlights of them were transferred to highly sensitive regions of the body as well, such as the brain [63]. So, the challenge still concerns clinical and diagnostic uses, but this does not mean that these types of research fields are afforded a multidisciplinary point of view toward multimodal analysis and high-impact solutions accompanied with fundamental knowledge at the same time. Just to finish, it is worth mentioning that other particles such as PET/NIRF/MRI triple-functional iron oxide nanoparticles [64], gold nanoparticles and quantum dots for bioimaging were generated using different forms of energy signaling [65]. Organic matter with additional properties, in particular from structures based on carbon with highly conjugated electronics, such as graphene, carbon nanotubes (CNTs), and further allotropes, were found by radiotracing methodologies within living animals [66]. Considering further multimodal and hybrid structures, the coupling of treatments could afford multifunctional approaches the ability to detect, track and deliver treatments whilst it is possible to monitor the targeted biostructure in place. Soft materials and other energy modes could be involved. In this regard, recent developments focused on the innovation of graphene oxide nanoplatforms for targeting tumor diagnosis coupled with treatments affording multiple tasks and functions. In this manner, chemotherapy–photothermal–photodynamic synergy and improved simultaneous performances were achieved [67]. Thus, colloidal formulations and the evaluation of modes of administration are of interest as well.

So, the nanoscale is still under evaluation, and the enhancement variables involved depend on the applications as well. Therefore, the use of nano-formulations is in focus and involves multimodal approaches considering current advanced instrumentations that combine targeted nanoparticles with radioisotopes in order to track tracer distribution within tissues in vivo. Thus, PET, and Single-Photon Emission Computed Tomography (SPECT) could be mentioned as modes allowing the non-invasive real-time monitoring of agents within the body with a higher quantity of information generated [68]. In this regard, various hybrid nanomaterials could be applied for the different energy modes required [69]. And it should be highlighted that not many have been tested and evaluated on real sample applications. So, there is huge potential for research and development in this area. Radiolabeled nanoparticles represent a new class of agent with great potential for bioimaging and biophotonics with future clinical uses. So, new optical approaches considering new architectures incorporating many energy modes could provide varied signaling. This is the first perspective, but when photons, electrons and matter interact, there are non-predicted behaviors, an aspect of the new knowledge mentioned previously, that are of high interest as well. Finally, it is remembered that less-invasive techniques and faster detection, diagnosis and delivery of treatments depending upon need are always preferred.

## 4. Advances in Hybrid Confined Nano, Quantum, and Radionuclide Architectures for Multimodal Imaging

Looking at multi-functional nanomaterials, it is possible to design varied combinations of compositions of materials to produce different functional components with defined roles to achieve a final targeted application. Moreover, it is possible to propose synergic systems for the final use as well. So, innovation is a challenge that depends upon need. However, in all cases of confinement within the nanoscale, the capability to collectively consider emitters, quantum non-classical physics, and further radiation, which causes matter sources to interact, and to modify their initial electronic properties is highlighted. Thus, new energy modes as well as those for simultaneous delivery could be generated. In this way, organic materials, through chemical variation, are permitted to be a part of the whole nanostructure, from simple linkers to support material, to add particular electronic and luminescence properties to inorganic materials such as metals, semiconductors, and quantum emitters. It is possible to propose new prototypes and approaches for the generation of multimodal imaging systems in the context of research and applications, such as translating biophotonics to clinical assays [70]. For example, that current developments incorporating fluorescence within multimodal imaging platforms for high-throughput morpho-dynamics and spatial biology are underway [71]. Moreover, researchers are seeking enhancements and new light–matter properties to improve imaging and innovation for bioimaging applications, which could be highlighted as a translational trend towards new theranostic approaches. Thus, tumor-targeted radionuclide nanomedicine for clinical translation shows advances towards precision therapy [72]. In these types of approaches, it is shown how important the cargo and confined systems are in protecting the sources of signaling that just arrived at the desired tissue. Moreover, the capability of multifunctional approaches at tiny sizes showcases the versatility of nanotechnologies, ranging from colloids to single nanoarchitectures interacting on individual cells. In this regard, dual-targeting albumin hitchhiking for improved tumor accumulation was shown in the context of the use of radionuclides within the nanoscale. The stimulus-responsive chemistries that synchronize isotope release with tumor microenvironmental regulation effects were evaluated and optimized. Therefore, multifactorial events that could affect the targeted therapy were shown. Moreover, other designs contemplated the incorporation of guided approaches using specific vectors and carriers accompanied with further materials, such as nanoporous nano-metal–organic frameworks labeled with radionuclides [73].

In this manner, it could be shown how the design and synthesis of varied nanoarchitectures could lead to different insights and impacts focusing on different challenges and needs in this multidisciplinary research field. Therefore, multifunctional hybrid organic/inorganic nanomaterials for tuning enhanced non-classical light emissions, drug delivery applications, molecular and biomolecular detection, signal waveguiding, and other nanotechnological applications are still at the cutting edge of research, developments and technology transfers. So, in this case, it is important to note that the targeted objective is focused on the development of a modified signal, i.e., an enhanced one. Also, new electronic densities and matter physics with new energy modes and properties, such as those known from nominated metamaterials, could be generated.

So, for the incorporation of nanoplasmonics and electromagnetic fields, inorganic components should be involved to produce spontaneous emissions and associated studies. Therefore, light–matter interactions, photonics, and quantum and radionuclide emissions could interact with further optical matter to generate enhanced spontaneous emissions, such as those developed by Purcell et al. [74]. In this manner, advanced developments within confined micro- and nano-devices [75] are accompanied by a huge number of studies related to high-electromagnetic-energy fields generated from metallic surfaces within variable lengths of intervals. Spontaneous emissions of electromagnetic fields were studied in the beginning by Purcell et al. within the micro- and macro-scales; in 1952, they were awarded the Nobel prize in Physics for nuclear magnetic precision measurements [76]. These studies afforded quantum developments and nanoplasmonics. The near field of electromagnetisms was described by the dense electron gas model using the Maxwell equations [77], and electronic oscillations of metallic surfaces were explained by the Mie theory [78]. The Mie theory solves the Maxwell equations, permitting further advanced experimental studies within the nanoscale and beyond [79]. Thus, variations in high-intensity electromagnetism fields from tunable nanoplasmonic surfaces were experimentally shown, with varying metals and nanochemistries involved. In this manner, the generation of variable emission wavelengths and applications in light–matter interactions were actually studied. Thus, different nanomaterials showed unique spectroscopical properties along the entire electromagnetic spectrum. It is noted that metallic nanoparticles act as versatile platforms and templates to produce designs and properties based on new nanomaterials and metamaterials. In this context, the tuning and modulation of optical active materials involving different levels of chemistry and physics could be involved. In this manner, life science applications and fundamental studies showed the importance of bioconjugation techniques using varied spacer and chemical reactions as strategies to develop biophotonics, biotechnology and nanomedicine systems. In a similar manner, it could be noted that photonics and other nanotechnology developments toward hard materials using chemical reagents, passivation agents, stabilizers and more targeted functions permit the modification of sizes, shapes, and topological phenomena. Thus, new potential physical and chemical properties with potential technology transfers could be discovered. This capability to tune radiation is affected by the presence of bioconjugates, which indicates the need for further studies affording bioconjugations after the development of specific targeted nanoemitters.

Moreover, other types of nano- and microparticles based on organic materials are micelles and vesicles, which have shown huge potential for application within the life sciences [80]. In general, as controlling compositions, they are well accepted and biocompatible for drug delivery applications within biorelevant conditions [81]. Researchers recently reported the dual delivery of different properties from colloidal lipidic nanoparticles such as Carvacrol-loaded liquid crystalline nanoparticles based on phytantriol and Peceol^®^. This combination produced a novel nanoplatform for controlled drug delivery and fluorescence generation [82]. But, in this context, it is highlighted that the confinement of emitters or optically active radiative matter occurs within soft matter. The optically active media are different and could be modified by chemical derivatization. Therefore, organic chemistry and synthesis should be involved in their modification, such as by focusing on lipids from the head to the tail, depending on the needs of the new reagents.

In this regard, multiplexed imaging in cancer diagnosis showed advances with the simultaneous use of two or more techniques incorporating contrast reagents, accompanied with different carriers and matter properties [83]. Thus, a combination of different energy emissions was proposed in order to combine various signaling and multicolor approaches from different instrumentation. It was possible to record a higher quantity of data generated from the same hot spot under study. The multiplexed imaging strategies aim to overcome the limitations of individual conventional techniques by producing different colors and energies while focusing on the same detection event. For this reason, there are actually many existing imaging technologies that are broadly categorized as either mainly anatomical or mainly molecular. However, in seeking faster and earlier diagnoses as well as intending to track new treatments, the proposal of multiplexed approaches was optimized and accompanied with proper methodologies to incorporate different emitters. For example, one could mention the already developed and applied multiplexed imaging technology of combining anatomically resolved CT with physiological imaging data of PET. Here, both provide functional and anatomical information [84]. For oncology, the combination PET and CT was used for cancer screening and diagnosis, involving the monitoring and regulation of the therapy based upon need. So, by combining multimodal optical setups, more powerful clinical methods are afforded [85]. In this context, further multiplexed imaging of radionuclides was later proposed [86]. Multiplexed radiotracers and controlled varied combinations of them could vastly improve the comprehension of cellular dynamics and metabolic processes. The use and clinical feasibility of multiplexed radionuclide imaging strategies involved a logical scale up in the application, incorporating human patients monitored in real time. Therefore, real-time imaging of multiple simultaneous biological processes with a lower time of instrumental exposure to patients was achieved. These recent biological results point to new therapeutic interventions and personalized medicine at the cutting edge of the ongoing fundamental and applied translational clinical research. Therefore, targeted goals such as faster diagnoses, less-invasive methods, reduced side effects, improved efficacy, and optimized treatments were intended to be evaluated and developed. So, these perspectives are expected to generate multiplexed, multimodal approaches to produce alternative methods. In this manner, the use of multiplexed nuclear imaging such as standard clinical assays could positively modify the management of diagnoses and treatments with the comprehensive management of big data information. Faster and early decisions are possible, as is tracking the development of treatment. In this context, and in a similar manner as radio-nuclei tracers’ solubility, accessibility to targeted cells and tissues requires bioconjugations and conjugations with different molecules depending on applications within confined spaces.

**Figure 6:**
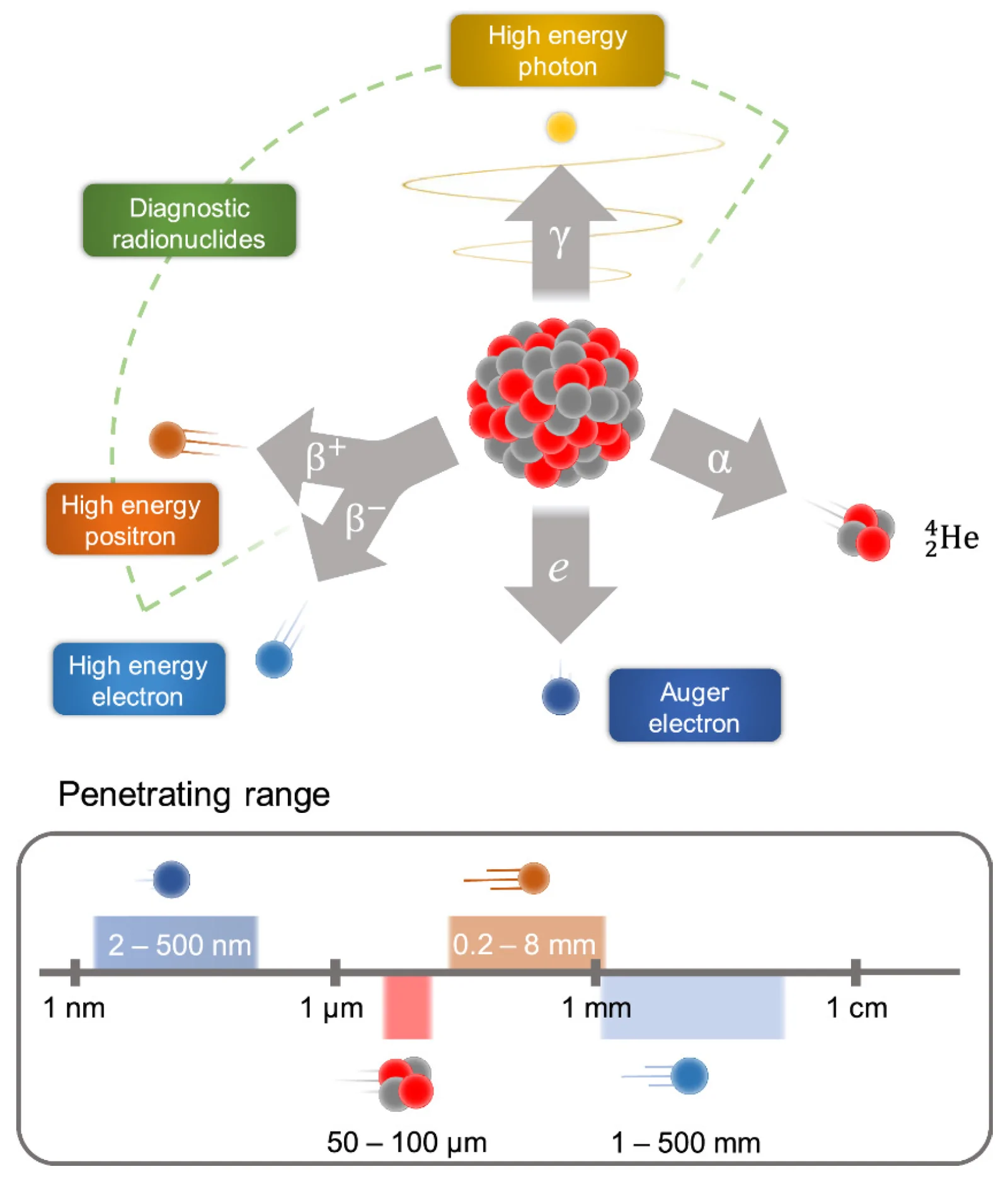
Types of radioactive decay with a demonstration of the soft tissue penetration range. Published with the permission of the authors; citation: [87] Oleksii O. et al., A. S. Timin, 2019, Journal of Nanobiotechnology, Springer Nature.

From another point of view, it could be highlighted that the challenge is to deliver multimodal energies from tiny nanoplatforms in close proximity to cells, and even embedded within membranes. These new bioconjugated nanomaterials would ideally provide different forms of chemical modification, as well as molecular and tuned nanostructural incorporation of various sources of labelers, tracers, and pharmacophores, joining switch-on/off systems. This proposed versatility alongside their intrinsically different properties in comparison to free drugs could play a role in different, new studies and optically active nanoplatforms, where radiotracers are incorporated and potentially further conjugated with even more optics. The different rates of radionuclide decay and variable deep penetration within soft tissues are highlighted, depending on the energy involved in the multiplexed sources applied (Figure 6) [87]. From this perspective, the further emitters mentioned could be related to different types of particles and associated with different kinds of physics, such as those accompanying bosonic and photonics materials. New methods considering biophotonic techniques and methods are highlighted in the next generation of instruments [88].

New bioinformatics strategies are required for the manipulation of huge quantities of information from imaging, as well as to discuss the need for libraries of targeted information, such as types of tissues and biomolecular profiles. Thus, additional considerations should be involved in bioassays, such as multifactorial analysis, machine learning, and artificial intelligence (AI). New technologies considering the bioanalysis and delivery of varied multimodal energy modes within different intervals of time could be proposed. In this direction, the integration of artificial intelligence for precision medicine and more accurate dosimetry is mentioned as an example. The combination of therapeutic approaches using targeted radionuclide therapy with a synergistic application of chemotherapy and immunotherapy stimulated externally by beam radiation showed promising, positive results in refractory cancers. In this manner, PET (Positron Emission Tomography), Single-Photon Emission Computed Tomography (SPECT) imaging, and therapeutic applications using alpha and beta emitters such as 225Ac and 177Lu are mentioned. These prototypes were combined and passed clinical trials that afforded them approval as current clinical agents and formulations. In this manner, their clinical applications for neuroendocrine tumors and metastatic castration-resistant prostate cancer were reported [89].

Finally, there is a need to develop real multimodal technologies with multifunctional nanoplatforms that significantly enhance cancer diagnosis and treatments at the same time. This means that phenomena and functions are performed. The examples show that by incorporating different forms of real signaling, radiation–matter interactions could not always be achieved between them, and combinations of different properties could be optimized. For example, gold-based nanoparticles, such as peptide-functionalized nanostructures and PEG-coated nanorods, offer improved tumor targeting; multimodal imaging, including photoacoustic and fluorescence techniques, accompanies effective photothermal therapy [90]. These closing examples could highlight many combinations of confined materials interacting with further optically active matter to tune emissions. In addition, bioconjugations and targeted interactions involving immunology acted as directional carriers towards the controlled delivery of emissions and even drugs based upon need.

## 5. Conclusions and Future Perspectives

As was shown, there are many well-performing imaging techniques and methodologies available for early diagnosis and precision medicine uses. However, at the same time, there is increasing interest in tuning multimodal approaches considering only the instrumentation. In addition, the miniaturization of technology has opened new challenges where reduced sizes should show high sensitivity and specificity, including devices, chips, and smart platforms with varied sizes. In this regard, we highlight the development of nanoscale and quantum technology to improve, participate in, or incorporate signals for varied optoelectronics applications, such as the generation of imaging. The control of these scales could be used in the development of the instrument or as part of a required important reagent, labeler, or tracer for imaging. However, there are still existing challenges to overcome in the different imaging techniques for targeted diagnoses. Most of them are related to resolution, specificity and time-consuming factors, the level of invasiveness of procedures, data managing, analysis, AI applications, the development of computing systems, the software accessible to different persons with varied skill levels, etc. In this regard, fundamental and applied research is providing many insights. At the same time, new reagents and nanostructured materials based on a new basis and concepts [91,92,93,94] could be proposed, contemplating labelers, standards, tracers, and available new molecules and nanoparticles ready to be used for further developments. In this context, fundamental and applied research could be combined with patent generation, driving technological insights towards technology transfers.

In the search for multimodal approaches, there is a lack of developments where many energy modes are incorporated within reduced sizes, from molecules to quantum dots and nanostructures. So, the need to propose new hybrid nanoarchitectures with high stability accompanied by strong signaling is highlighted. Thus, from the combination of different materials, it could be possible to generate multimodal imaging with just one procedure applied to the biostructure, with this depending on the optical instrumentation being excited in the same place and at the same time. In this manner, different levels of information could be achieved depending upon need. In addition, fundamental knowledge related to energy–matter interactions could be produced. So, the interest is broad and it contemplates many aspects focusing on science as well technology. In this context, the importance of two components at different levels in the bottom-up development of the technology is highlighted, but with the same importance considering similar contributions, such as nano-optics and optical instrumentation, as, for example, the contributions showed by combining the varied PET radiotracers, chemistry and nanoscale involved.

**Figure 7:**
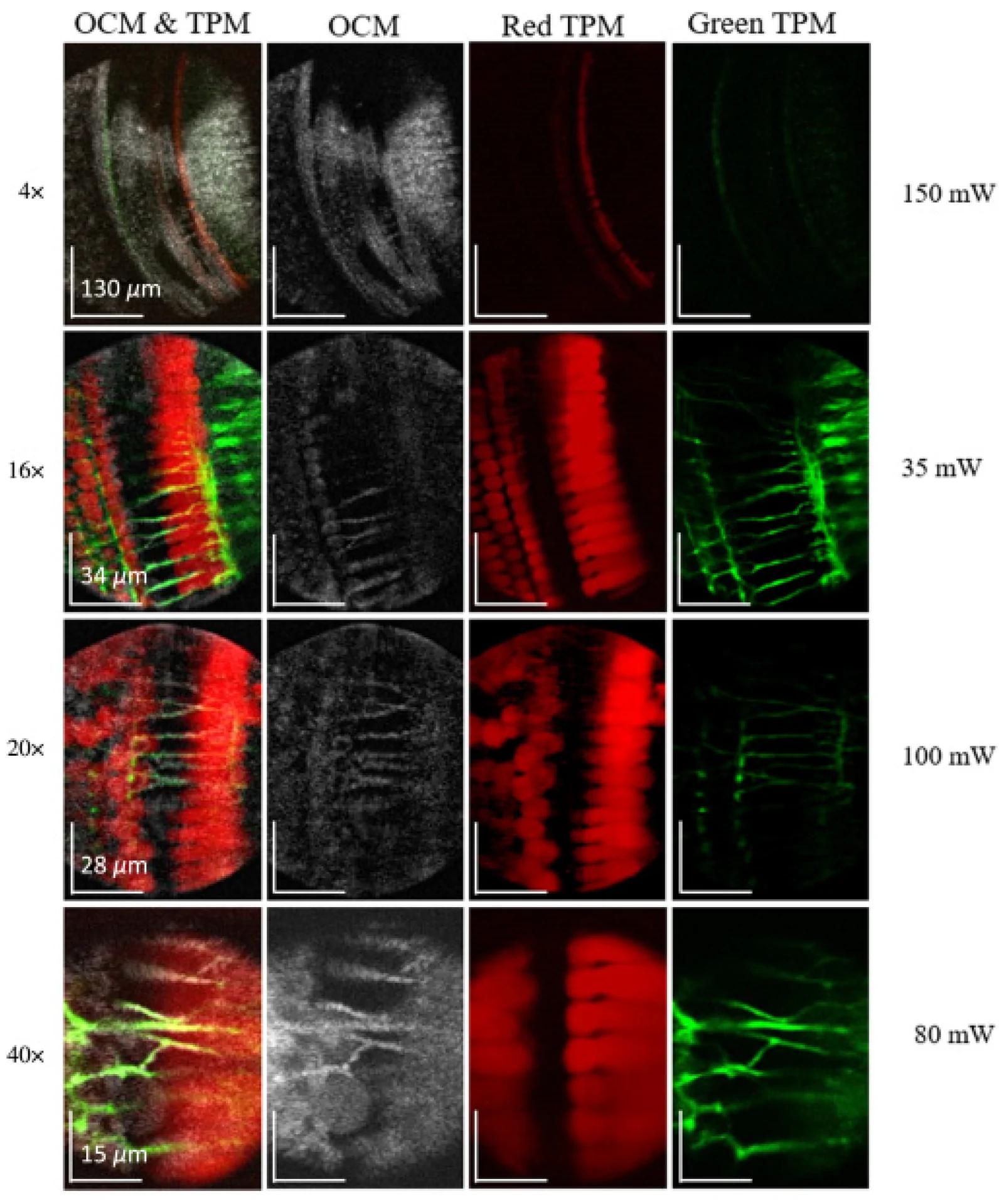
Representative images of an excised cochlea taken from a z-stack where the tissues of interest were in the focal plane of each objective. Each row corresponds to a different objective, as labeled, and each column corresponds to the image type labeled at the top of the column. The far-left column demonstrates the colocalization of both fluorescence channels with the corresponding OCM image. Representative olivocochlear (efferent) nerve fibers are indicated with arrows, inner hair cells are indicated with IHC, and outer hair cells are indicated with OHC. Labels on the right-hand side of the figure indicate the average power of the TPM excitation used to get the fluorescent images. Published with the permission of the authors; citation [95]: Clayton B. Walker et al., Brian E. Applegate, 2025, Journal of Biomedical Optics, SPIE.

In this regard, the incorporation of OCM and multi-colored fluorescence within new optical setups, considering both techniques and applying new methods to achieve the respective signaling afforded to higher resolutions of living mouse cochlea, was shown (Figure 7) [95]. It was shown how one of the most highly sensitive techniques afforded a non-expected colored resolution accompanied by a high degree of detail provided by the coupled second technique. This is the concept of the multimodal approach for enhanced results and analysis showed in a real sample. In brief, the fluorescence signaling showed the two fluorescence channels and OCM overload to confirm that OCM can be used to find the precise location of a region of interest in the tissue depending upon need. Moreover, the OCM column further confirmed that it is possible to resolve important landmark structures without the aid of TPM. Therefore, the details of nerve fibers can be clearly seen, and controlling high-NA objectives performs well in this regard. This combination of signaling showed potential unused available information from the molecular scale to nano- and micro-sized and higher tissue scales. So, this could be considered the beginning of biomolecular fluorescence detection and diagnosis within multiplexed and multimodal imaging techniques.

In this context, it is mentioned that all three high-NA objectives assayed were able to clearly visualize the important fluorescent tissues to complete the high-NA task of localizing and monitoring dendrite swelling. And, for the neophytes, it is mentioned that in microscopy, Numerical Aperture (NA) quantifies a lens’s light-gathering ability and resolving power, indicating how finely it can distinguish details. It can be calculated and it depends on the refractive index of the medium (air, or oil) and the angle θ as half the objective’s angular aperture. A higher NA means a better resolution (sharper images) and brighter images, often requiring immersion oil for NAs above 1.0 to capture more light and detail, especially at high magnifications. These are notes to open further discussions about how photonics emissions from fluorescence-labeled tissues could be collected. Moreover, it should be known that the laser beam, if considered as a fine slide, could involve micrometer lengths with varied shapes where the focus could cover many emitters incorporated in a targeted hot spot.

**Figure 8:**
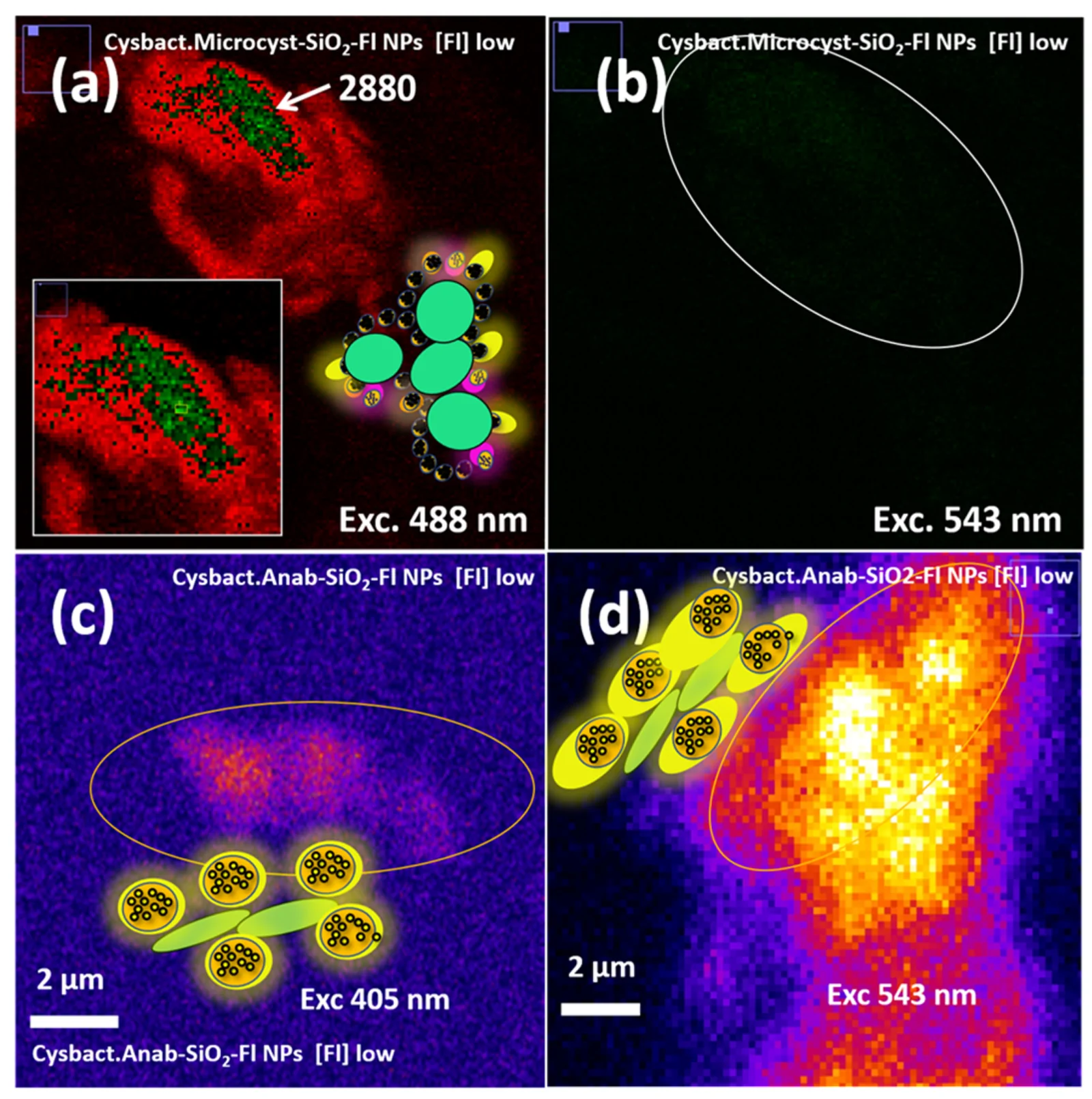
Cyanobacteria labeling with fluorescent silica nanoparticles: (**a**) laser fluorescence microscopy imaging of cyanobacteria Microcystis-labeled with SiO_2_–Fl nanoparticles with excitation at 488.0 nm. Inset images of Confocal Laser Fluorescence Microscopy of nano-biostructures (Microcyst -SiO_2_-Fl-); (**b**) laser fluorescence microscopy imaging of cyanobacteria Microcystis-labeled with SiO_2_–RhB nanoparticles with excitation at 555.0 nm. (**c**) Nano-bioimaging of Anabaena sp. applying cargo-loaded silica nanoparticles with a low Fl concentration. Laser excitation at 405.0 nm, and (**d**) 543.0 nm laser excitations. A Lookup Table (LUT) set was fired to adjust image brightness and contrast. Reprinted with the permission of the authors; citation: [93,97] Bracamonte et al. 2020 and 2025, RSC Adv, and Discovery Chem, respectively.

In addition, biophotonic insights recently showed various reports related to light delivery from confined silicon-based nanoemitters with incorporations of donor–acceptor laser dyes for enhanced Fluorescence Resonance Energy Transfer (FRET). Thus, controlled enhanced FRET silicon-based nanoplatforms that were grafted on unicellular and poly-cellular organisms such as Microcystis and Anabaena species were achieved (Figure 8). In this manner, it was possible to tune emissions from a single nanoemitter as well as dimeric forms with even higher, enhanced emissions based on additional effects associated in part with dimeric energy resonances between particles, such as those developed from scattering lasers [96]. Therefore, non-classical light was delivered via FRET towards Photosystems I and II of both biostructures. In this manner, it was able to switch lasers and activate Bio-FRET pathways, resolving different nanopatterns and bioimaging depending on the donor–acceptor targeted. The higher emissions from free single nanoemitters were recorded by applying the shorter excitation wavelength dye as an energy donor based on FRET; however, the coupling of PSI and PSII governed the Bio-FRET pathways and synthetic Bio-light delivered. In the presence of *Microsystis* sp. [97], the shorter laser excitation produced a higher and enhanced resolution of bioimages in the absence of an energy acceptor because quenching phenomena occurred when the light arrived at PSII; however, in the absence of the acceptor energy dye, it was possible to achieve higher-resolution and quality bioimaging (Figure 8a), while towards longer excitations via FRET or by directly applying longer laser excitations and energy acceptor dye excitations, quenched bioimaging was produced (Figure 8b). Then, by applying dimeric silicon-based nanophotonics systems on *Anabaena* sp. crossref.93 [93] in the presence of only the energy donor, as stated previously, the opposite effect was generated, tuning the excitations and coupling with PSI and II as well (Figure 8c,d). It was highlighted that improved resolutions were even achieved by applying shorter laser excitations, such as 405 nm, to the lower energy delivered and partially coupled FRET as well as Bio-FRET.

Thus, the light delivered from the nanoscale passed through biostructure-targeting acceptors and emitting photosystems with a selectivity based on the excitation/emission wavelengths as well as other parameters considered in the development and optimization of nanoemitters. In this manner, further capabilities are still being studied in relation to the spatial resolutions, bioconjugations, targeted uses, and generation of optical and electronic information correlating different states of biomatter involved in biological events, metabolism, functions and modifications.

Actually, it is known that multimodal optical biosensing for precision medicine and healthcare is at the cutting edge of technologies currently under development based on new research reports, publications, books and technologies on the market [98]. The concept of multimodal imaging has so far exceeded expectations, focusing on appropriate combinations of energy modes supported by advanced optical technology. Thus, high-impact neurophotonic insights were reported, focusing on multimodal optical imaging and modulation with simultaneous electrophysiology through Smart Dura in non-human primates [99]. It was applied to skull chips to track electric signaling from micro-sized hot spots with neural cell activity coupled to three-photon images generating 3D volumes of vascularized brain tissues.

These capabilities and the different colors generated, depending on the applied lasers and energy modes, with usable resolutions and information are expected to be developed for translational clinical biophotonic techniques and methods. In this context, new challenges emerge, such as biocompatibility and environmental constraints, that should be involved in the research in advance or at the same time [100,101]. Coupling these phenomena with radionuclides could support a lot of important research, from prototyping to applications in the detection of cancer cells, the anomalous growth of tissues and the development of new and alternative theranostics where diagnoses followed by effective therapies are expected to be applied. Moreover, silicon-based nanomaterials combined with radionuclides could be tuned as well. Many research developments were recently reported for non-porous silica-based platforms. An advancement towards clinical applications was shown for therapeutic and theranostic systems incorporating radionuclides [102]. An emphasis on the design of optical active platforms for imaging-guided radiotherapy and radiosensitization was shown. Thus, organosilica nanoparticles are tunable, optically active platforms with excellent dialectical properties and versatile chemical structures in combination with organic chemical compounds and beyond, including quantum and nanoemitters. Moreover, the mentioned versatility of these optically active materials permits the control of architectures incorporating new optical spaces to work on, such as holed and mesoporous nanomaterials. This versatility leaves free cargo holes with interstitial, tunable silica matter and nanosurfaces to design new nano-prototypes with different properties compared to solid nanoplatforms [103].

So, radionuclides, optical imaging and fluorescence are currently in focus to develop new multimodal energy modes delivered through space and time with different technological interests [104,105,106]. Finally, remote systems could be developed to control the delivery of energy modes based on varying physical and chemical strategies. Thus, photo-switchable materials activating responses through different strategies such as optical, chemical and electrochemical stimulations could be ascertained. In this direction, it should be noted that three simple examples with complex designs and synthetic routes achieve the stated simplicity. First, it could be applied to the chemical reactions involved in the delivery of supramolecular complexes in the context of potential drug-delivery applications [107]. Then, it could be applied to the controlled reversible modification of inorganic phosphors based on chromism reactions. Thus, traditional luminescence modulation techniques, such as core–shell structure design and energy transfer-related luminescent ion regulation, achieved reversible luminescence modifications [108]. Finally, the last example, related to the optical stimulation of electrochemical processes associated with metal dissolution, is highlighted, which involves hollowed nanostructures with new modes of resonant electromagnetic fields, such as enhanced plasmonic properties. The fabrication of a reversible shape and plasmon tuning in hollow Ag/Au nanorods by laser stimulation could be highlighted [109]. In this manner, the high intensity of bimetallic nanorods was enhanced by resonant electromagnetic fields produced within the hollowed nanorods. It is important to mention that the incorporation of luminescence within the X-ray range could be considered as extreme molecular and functional sensitivity, suitable at this moment for further, deeper studies. Therefore, X-ray and luminescence multimodal imaging is a powerful analytical technique that combines deep-tissue, high-resolution structural mapping with other sources of fluorescence, as well as bioluminescence and radioluminescence [110]. In addition, the combination of X-ray transmission, fluorescence, and other modes such as scattering tomography provides simultaneous energy modes for image reconstruction of tissues. This approach permitted high-resolution tissue structure images, quantitative imaging of high-Z element concentrations, and electron density distributions.

As stated, the final properties and applications are always highlighted; however, it should be noted that to achieve these levels of clinical translations, fundamental research should be developed first. An open opportunity is highlighted to study the tuning of multimodal energy modes where signaling could be modified and new optical insights could be incorporated. These are just mentions of further variables that could be involved in future studies for the next generation of multimodal imaging [111].

In conclusion, fundamental studies are in progress, aimed at shaping new technologies and optical setups considering higher performance in tiny and miniaturized instrumentation with the capability to manage a higher quantity of information. Thus, this leads towards the current challenges associated with furthering these themes and topics, such as critical thinking, AI during the generation of imaging, and decisions involving diagnoses. All of this should involve the different challenges associated with multifunctional imaging nanoplatforms, comprising acceptable biocompatibility, the required pharmacokinetics, and radiolabel stability over time in the absence of (or with diminished) toxicity, affording a big step towards clinical translations, trials involving bioethics regulations, and biohazardous procedures [112].

## Data Availability

No new data were created or analyzed in this study. Data sharing is not applicable to this article.
